# Cross-Cultural Adaptation and Psychometric Testing of the Brazilian Version of the Self-Care of Heart Failure Index Version 6.2

**DOI:** 10.1155/2013/178976

**Published:** 2013-09-15

**Authors:** Christiane Wahast Ávila, Barbara Riegel, Simoni Chiarelli Pokorski, Suzi Camey, Luana Claudia Jacoby Silveira, Eneida Rejane Rabelo-Silva

**Affiliations:** ^1^Graduate Program, School of Nursing, Federal University of Rio Grande do Sul, Rua São Manoel 963, Bairro Rio Branco, 90620-110 Porto Alegre, RS, Brazil; ^2^Cardiology Division, Heart Failure Clinic, Hospital de Clinicas de Porto Alegre, Porto Alegre, RS, Brazil; ^3^University of Pennsylvania School of Nursing, Philadelphia, PA, USA; ^4^Statistics Department, Mathematics Institute, Federal University of Rio Grande do Sul, Porto Alegre, RS, Brazil; ^5^Graduate Program in Epidemiology, Federal University of Rio Grande do Sul School of Medicine, Porto Alegre, RS, Brazil

## Abstract

*Objective*. To adapt and evaluate the psychometric properties of the Brazilian version of the SCHFI v 6.2. *Methods*. With the approval of the original author, we conducted a complete cross-cultural adaptation of the instrument (translation, synthesis, back translation, synthesis of back translation, expert committee review, and pretesting). The adapted version was named *Brazilian version of the self-care of heart failure index v 6.2*. The psychometric properties assessed were face validity and content validity (by expert committee review), construct validity (convergent validity and confirmatory factor analysis), and reliability. *Results*. Face validity and content validity were indicative of semantic, idiomatic, experimental, and conceptual equivalence. Convergent validity was demonstrated by a significant though moderate correlation (*r* = −0.51) on comparison with equivalent question scores of the previously validated Brazilian European heart failure self-care behavior scale. Confirmatory factor analysis supported the original three-factor model as having the best fit, although similar results were obtained for inadequate fit indices. The reliability of the instrument, as expressed by Cronbach's alpha, was 0.40, 0.82, and 0.93 for the self-care maintenance, self-care management, and self-care confidence scales, respectively. *Conclusion*. The SCHFI v 6.2 was successfully adapted for use in Brazil. Nevertheless, further studies should be carried out to improve its psychometric properties.

## 1. Introduction

Over the last few decades, treatment of heart failure (HF) has been optimized substantially through the advent of new therapies that have improved morbidity and mortality outcomes. These therapies, combined with nonpharmacological management strategies, have provided several benefits for patients, particularly in quality of life and rate of hospitalization due to decompensated HF [1, 2].

Within this context, nonpharmacological management, which encompasses a continuous process of patient education and development of self-care skills, has been widely studied and shown to be beneficial in the achievement and maintenance of clinical stability [2]. Among available self-care strategies, the multidisciplinary approach appears to be associated with the greatest benefit over time, improving quality of life, reducing readmission rates, and cutting health care costs [3, 4]. 

Self-care in HF is defined as a naturalistic decision-making process used to maintain physiologic stability (self-care maintenance) and respond to symptoms when they occur (self-care management) [3]. During this process of systematic patient education, skills such as interpreting sodium levels in nutrition facts labels, monitoring symptoms of HF deterioration, and developing a setting-specific exercise plan (tactical skills), as well as adhering to dietary restrictions and taking medications in unexpected situations (situational skills), which are required for implementation of self-care by patients or their caregivers, come to the fore [5, 6]. 

Instruments and scales for assessment of self-care were developed in response to the need to evaluate the effectiveness of self-care guidance provided to patients and to measure the impact of self-care on clinical endpoints [7, 8]. However, the extent and maintenance of treatment adherence in chronic disease involves highly demanding self-care behaviors. Recent studies have demonstrated some limitations in the development and validation of self-care instruments for patients living with chronic illness [9]. One such limitation is the need for these instruments to be employed in clinical studies, so as to establish their actual applicability and effectiveness in the clinical setting of patient followup [10].

Few investigators have proposed validated, user-friendly instruments designed specifically for patients with HF [11]. Within this perspective, a team of US nurses developed the self-care of heart failure index (SCHFI), currently in version 6.2, which covers all essential aspects (early recognition of signs and symptoms of decompensation, evaluation of the relevance of these signs and symptoms, decision to take action in response to signs and symptoms, implementation of a treatment strategy, and evaluation of the effectiveness of the implemented strategy) involved in self-care of HF. The SCHFI scale can be used to assess self-care behaviors in three domains: maintenance, management, and confidence. This scale allows assessment of patients' ability to recognize the signs and symptoms of HF decompensation, of the decision making process, and of the confidence in performing self-care actions [12]. 

The relevance of this study to clinical practice lies in its objective, which is to validate an instrument that assesses self-care in its different dimensions and, especially, identifies which dimension of self-care is impaired. This identification of barriers and challenges faced by patients allows planning and implementation of customized strategies. These strategies may improve patient adherence, knowledge, and self-care skills and, consequently, help patients achieve clinical stability. Within this context, the aim of this study was to adapt and evaluate the psychometric properties of the Brazilian version of the SCHFI v 6.2.

## 2. Methods

### 2.1. Study Design and Participants

This methodological study was conducted at a large teaching hospital in Southern Brazil. All adult patients with a diagnosis of HF (systolic or diastolic) who had received periodic followup at the HF clinic for at least 6 months and had attended at least one appointment with the heart failure nursing team during the year preceding the study were eligible for participation. We excluded patients with cognitive deficits that might hinder understanding of instrument items, based on a recorded history of dementia or other neurological conditions as well as on the investigator's assessment of participant orientation to time and place before completion of the instrument. Also, participants were considered to have some degree of cognitive decline if they had difficulty in answering any survey instrument items or required additional explanation after answering the questions. Patients with chronic obstructive pulmonary disease were also excluded (due to the difficulty of distinguishing COPD-related dyspnea from HF-related dyspnea), as were those with motor impairments or locomotor disturbances which would hinder assessment and grading of some items of the scale (e.g., items 4 and 7, which concern physical activity and exercise). The self-care management subscale was only administered to patients who had experienced signs or symptoms of decompensation in the one month preceding the study. 

### 2.2. Self-Care of Heart Failure Index Version 6.2 (SCHFI v 6.2)

The SCHFI v 6.2 scale, developed in the United States, comprises 22 items divided across three scales: self-care maintenance (10 items), self-care management (6 items), and self-care confidence (6 items). Answers for each item range from “never or rarely” to “always or daily” in the self-care maintenance scale, “not likely” to “very likely” in the Self-Care Management scale, and “not confident” to “extremely confident” in the Self-Care Confidence scale. Total scores for each scale are standardized to range from 0 to 100; higher scores reflect greater self-care ability, and self-care is considered adequate when all scales have scores of 70 or higher. The authors recommend that each scale should be administered separately and that the Self-Care Management scale should be administered only to patients who have experienced dyspnea and lower extremity edema within the last one month [12]. 

The reliability of the original scale was assessed by means of internal consistency, with Cronbach's alpha coefficients of 0.55, 0.59, and 0.82 for the maintenance, management, and confidence scales, respectively. Confirmatory factor analysis yielded factor loadings with absolute values ranging from 0.09 to 0.60 (Maintenance), from 0.29 to 0.62 (Management), and from 0.49 to 0.79 (Confidence) [12]. 

### 2.3. Cross-Cultural Adaptation of the Instrument

Before the start of the cross-cultural adaptation process, we contacted the original author via email, seeking her consent for validation and use of the instrument in Brazil, which she promptly granted. 

The cross-cultural adaptation process consisted of the following steps, as recommended in the literature [13]: translation, synthesis, back translation, synthesis of back translation, expert committee review of the translated version, and pretesting. 

During the cross-cultural adaptation process, some changes were made to the wording of certain items, and some examples of daily routines were included in the interest of patient comprehension. In item 5, for instance, the wording “keep appointments” was replaced with the term “assiduously”; in item 8, the wording “Not take (one of your medicines)” was used instead of “Forget to take,” as patients in our setting often skip medication due to socioeconomic conditions and difficulty obtaining access to the health services rather than forgetfulness.

After convening the expert committee and seeking clarification of certain issues with the author of the original instrument, we had the *Preliminary Adapted Version* of the SCHFI v 6.2 available for pretesting.

### 2.4. Pretesting

Thirty patients were selected only for this stage. The mean time to completion of the three subscales was 8 minutes. No modifications were required after pretesting; therefore, the preliminary adapted version was kept unchanged as the final portuguese version, which was named *Brazilian Version of the Self*-*care of Heart Failure Index version 6.2*, or SCHFI v 6.2 (Brazilian).

### 2.5. Assessment of Psychometric Properties

Psychometric testing of the scale was carried out as recommended elsewhere in the literature [14], in a process consisting of the following stages: face validity and content validity (by expert committee review), construct validity (convergent construct validity and confirmatory factor analysis), and reliability (by analysis of Cronbach's alpha).

Assessment of face validity measured understanding and acceptance of the items of the scale, as expressed by a consensus among the members of the expert committee (two nurses with clinical expertise in the care of HF patients, a nurse with experience in the care of patients with heart disease who was also a teacher of Portuguese, a registered dietitian of the hospital outpatient HF clinic, a nurse with experience in the study methodology, the first author, and her academic advisor) and the study respondents (pretesting stage), with the chief purpose of assessing whether the instrument measured what it set out to measure [14]. Content validity was determined by a consensus of the expert committee as to the relevance of each instrument item for measurement of the parameters of interest. 

In this study, convergent validity was assessed using the previously validated *Brazilian Version of the European Heart Failure Self*-*care Behavior Scale*, or EHFScBS (Brazilian) [11], as a gold standard. The validated EHFScBS (Brazilian) scale consists of 12 questions within a single domain related to self-care behavior. The responses for each item range from 1, “I completely agree,” to 5, “I do not agree at all,” on a five-point Likert-type scale. The total score is obtained by adding all of the answers and can range from 12 to 60. Lower values are indicative of better self-care. The items concern various self-care behaviors of patients with heart failure, such as daily weighing, rest, contacting a health care provider, fluid restriction, diet, medication adherence, annual flu vaccinations, and exercise. Cronbach's alpha for the EHFScBS (Brazilian) was 0.70 [11]. 

Confirmatory factor analysis was performed to confirm the factor structure of the original instrument. 

The reliability of the *Brazilian Version of the Self-care of Heart Failure Index v 6.2* was verified by assessment of internal consistency (measured by Cronbach's alpha). 

The scale was administered to all participants by means of an interview, in a private room. On average, respondents took 8.2 ± 3 minutes to complete the scale.

### 2.6. Data Analysis

Continuous variables were expressed as means ± standard deviations. *P* values <0.05 were considered statistically significant. Statistical analyses were conducted in the statistical package for the social sciences (SPSS) 18.0 software environment. Confirmatory factor analysis was performed with the aid of AMOS 18.0 software [15]. In addition to the overall chi-squared statistic, several overall goodness-of-fit indices were employed to examine the fit of the factor model with the following “rule-of-thumb” cutoff criteria for well-fitting models: comparative fit index (CFI) > 95, root mean square error of approximation (RMSEA) < 0.05, and normed fit index (NFI) > 0.95 [15]. Cronbach's alpha coefficient was used to assess the internal consistency of the validated scale. 

## 3. Results 

### 3.1. Sociodemographic and Clinical Characteristics of the Sample

The study sample comprised 128 patients, the majority of whom were males (78.9%). Mean age was 61.4 ± 12.8 years, and most patients were retired (76.4%). The most common etiologies of HF were ischemic heart disease (41.4%) and hypertension (25%). The sample profile is described in greater detail in Table 1.

*Validity Testing*

### 3.2. Convergent Validity

A significant (*P* = 0.017), though weak, inverse correlation (*r* = −0.30) was found between overall scores for the Brazilian Version of the Self-care of Heart Failure Index v 6.2 and the Brazilian Version of the European Heart Failure Self-care Behavior Scale. On analysis of convergence between the five equivalent questions of the two scales, a significant (*P* < 0.001), moderate, and inverse correlation (*r* = −0.51) was found. An inverse correlation was expected as lower scores indicate higher self-care on the European heart failure self-care behavior scale.

### 3.3. Confirmatory Factor Analysis

**Figure 1:**
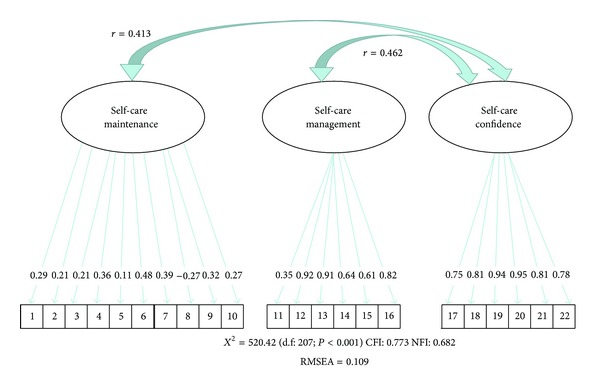
Confirmatory factor analysis. The figure shows the standardized loadings for the indicators of the latent constructs of the self-care maintenance, self-care management, and Self-Care Confidence scales. Numbers within the outlined boxes represent item numbers. Numbers outside the outlined boxes represent the factor loadings. The negative loading of item 8 is due to its reverse scoring. *r* represents correlations between the self-care maintenance and self-care confidence/self-care management and self-care confidence scales.

We used confirmatory factor analysis to test a three-component model in which the items of each component were those of the original instrument. In the original model, correlation between self-care maintenance and self-care management was not considered. Goodness-of-fit indicators for the tested model, including the three SCHFI v 6.2 scales, were as follows (Figure 1).

Most items had factor loadings with greater absolute values than those of the original model. These values ranged from 0.11 to 0.95 (Figure 1).

### 3.4. Internal Consistency

Internal consistency was assessed by means of Cronbach's alpha. The calculated coefficients were 0.40 for the self-care maintenance scale, 0.82 for the self-care management scale, and 0.93 for the self-care confidence scale.

### 3.5. Comparison between Mean Scores of the Brazilian Version of the Self-Care of Heart Failure Index v 6.2 and of the Original SCHFI v 6.2

The mean scores obtained with the Brazilian Version of the Self-care of Heart Failure Index v 6.2 were 57 ± 14.3 in the Maintenance scale, 47 ± 28.3 in the Management scale, and 58 ± 25.5 in the Confidence scale. All scores were lower than those obtained in the original study with a U.S. sample (Table 2). 

## 4. Discussion

This was the first Latin American study to conduct cross-cultural adaptation and psychometric testing of a scale for assessment of self-care by HF patients, namely, the SCHFI v 6.2. This scale assesses self-care abilities at each stage of the self-care process (maintenance, management, and confidence) in patients with heart failure. 

During the cross-cultural adaptation process, some terms and expressions were modified so as to facilitate understanding of scale items by patients and professionals who may wish to administer it, as well as to ensure cultural equivalence. Our communications with the author of the original scale allowed us to make minor modifications and add some real-world examples without affecting the substance of the scale. Testing of the Brazilian version of the European heart failure self-care behavior scale confirmed its convergent validity, both due to the statistical significance and to the strength of the inverse correlations. These results suggest that the component items of the two scales measure similar constructs [11].

In confirmatory factor analysis, we tested a three-component model in which the items of each component were those of the original instrument. Analysis confirmed this model had the best fit. 

As in the original instrument, the self-care management and self-care confidence scales had higher factor loadings than the maintenance scale [12]. The low factor loadings found for the self-care maintenance scale may mean that the defined questions do not reflect this construct accurately. For instance, item 5 (“How routinely do you… keep doctor or nurse appointments”) had a factor loading of 0.11, which means that only 1.2% of variation in this item is explained by self-care maintenance. This may be explained by the fact that study patients were treated under the auspices of the publicly funded unified health system, and may thus avoid missing appointments out of fear of losing access to care. Furthermore, the average number of visits per year is four at most, which further reinforces the importance of keeping all appointments, particularly as patients must refill their prescriptions. It bears stressing that adherence is self-reported, which may lead to some overestimation of assiduous medication use [16]. Furthermore, one may infer that adherence to medication use and attendance of appointments are behaviors that do not require major lifestyle changes and, therefore, are more easily achieved. Conversely, physical exercise, adherence to a proper diet, smoking cessation, and weight management are all directly related to lifestyle modifications that are difficult for patients to make, despite knowledge of the benefits of these practices [17].

Factor loadings for the self-care confidence subscale were exceedingly high (up to 0.95), which may suggest that these items are already explained by others. Items 19 and 20, which had the highest factor loadings (0.94 and 0.95 resp.), assess the importance of symptoms and the recognition of changes in one's health. These behaviors are quite similar and interconnected, as management of chronic illness does not depend solely on the knowledge acquired over time or through patient education programs. Self-care management also depends on personal resources, such as self-confidence, self-care skills, and the ability to recognize and manage changes in one's health.

In the present study, reliability was assessed by means of internal consistency, as measured by Cronbach's alpha. Coefficients for the self-care management and self-care confidence scales were adequate and similar to those found in the original study [12]. This suggests that the component items of the scale measure the same self-care attributes and are related to the overall scale as well as to self-care management and self-care confidence. In a validation study carried out in China, Cronbach's alpha values were only provided for the scale as a whole (22 items), which is no longer advocated [18]. 

Cronbach's alpha coefficients for the Self-Care Maintenance subscale were lower than those obtained for the two other subscales and lower than those obtained in the original study [12]. These values suggest that the component items of this scale warrant special attention and should be tested in different patient populations to ascertain equivalence. We believe that some items of this subscale (such as “do some physical activity” and “use a system… to help you remember your medicines”) do not actually reflect superior self-care skills. Some patients, particularly those with NYHA class II or III HF, refrained from physical activity due to exercise intolerance and development of symptoms on exertion. Furthermore, the use of a system to remember to take one's medications does not necessarily entail superior self-care. Due to the chronic nature of HF, many patients incorporate taking their medicines into their daily routines and do not need any system to help them remember.

Scores obtained for the self-care maintenance, self-care management, and self-care confidence scales of the Brazilian version of SCHFI v 6.2 were all below the defined cutoff for adequate self-care and were lower than those obtained in the original study and in later studies of adaptation and validation of the instrument for other cultures [12, 18, 19]. These findings are consistent with the existing literature, which suggests that approximately 50% of patients fail to comply with nonpharmacological measures (the behaviors assessed by the study instrument) [6]. The “Take an extra water pill” item of the Management subscale may also have contributed to lower scores, as the patient population from which our sample was drawn is not usually instructed in this practice. Another relevant factor concerned the item “Call your doctor or nurse for guidance” (*Contatar seu médico ou enfermeiro para orientação*). It bears stressing that the study was conducted at a public hospital, which has no 24-hour hotline to answer patient questions.

Self-care in HF still poses a challenge to providers, patients, and caregivers alike, but is an essential aspect of disease management. Hence, there is a pressing need for development of effective self-care strategies and, consequently, for assessment and measurement of changes in self-care behavior and of the self-care skills developed by patients.

## 5. Conclusion

The cross-cultural adaptation of a Brazilian Portuguese version of the SCHFI v 6.2 instrument followed the established process recommended in the scientific literature, which yielded a scale successfully adapted to the Brazilian reality. 

Convergent validity showed moderate correlation on comparison with equivalent question scores of the previously validated Brazilian European heart failure self-care behavior scale. Confirmatory factor analysis showed weak indices of CFA, and internal consistency testing demonstrated inadequate indicators for the maintenance scale alone. These findings suggest that further studies should be carried out to improve the psychometric properties of the SCHFI v.6.2. 

The relevance of this study and future investigations to clinical practice lies in the fact that validated scales can help nursing teams implement individualized patient management strategies, enabling constant evaluation of patients' self-care abilities, particularly with regard to the recognition of signs and symptoms of decompensation, symptom management, and confidence. 

### 5.1. Limitations

Some items of the self-care maintenance scale, such as “keep appointments”, “call your doctor or nurse,” and “use a system to help you remember your medicines” were not applicable to the reality of the study sample. Therefore, the presence of these items may have had an adverse impact on the internal consistency of the scale.

## Figures and Tables

**Table 1:** Sociodemographic and clinical characteristics of the sample (*n* = 128).

Variables		*n* (%)
Age, years*	61.4 ± 12.8	
Sex, male		101 (78.9)
Employment status, inactive		85 (76.4)
Educational attainment, years^†^	5 (4–8)	
Etiology of heart failure		
Ischemic heart disease		53 (41.4)
Idiopathic		32 (25.0)
New York Heart Association (NYHA) Functional Class		
I		37 (29.0)
II		65 (50.7)
III		26 (20.3)
Left ventricular ejection fraction (%)*	31.2 ± 12.7	
Duration of heart failure, months^†^	36 (17–58)	

*Mean ± standard deviation; ^†^median (interquartile range).

**Table 2:** Scores obtained with the Brazilian version of the self-care of heart failure index v 6.2 and with the original self-care of heart failure index version 6.2.

	SCHFI v 6.2 Brazilian version scores (*n* = 128)	SCHFI v 6.2 scores in USA (*n* = 130)
Self-care maintenance	57 ± 14.3	70 ± 14.3
Self-care management	47 ± 28.3	63 ± 22.6
Self-care confidence	58 ± 25.5	70 ± 16.2

All scores are expressed as mean ± standard deviation.
